# Diabetes Alters the Expression and Translocation of the Insulin-Sensitive Glucose Transporters 4 and 8 in the Atria

**DOI:** 10.1371/journal.pone.0146033

**Published:** 2015-12-31

**Authors:** Zahra Maria, Allison R. Campolo, Veronique A. Lacombe

**Affiliations:** 1 Department of Physiological Sciences, Oklahoma State University, Stillwater, United States of America; 2 Department of Mechanical and Aerospace Engineering, Oklahoma State University, Stillwater, Oklahoma, United States of America; 3 Harold Hamm Diabetes Center, University of Oklahoma Health Science Center, Oklahoma City, United States of America; Tohoku University, JAPAN

## Abstract

Although diabetes has been identified as a major risk factor for atrial fibrillation, little is known about glucose metabolism in the healthy and diabetic atria. Glucose transport into the cell, the rate-limiting step of glucose utilization, is regulated by the Glucose Transporters (GLUTs). Although GLUT4 is the major isoform in the heart, GLUT8 has recently emerged as a novel cardiac isoform. We hypothesized that GLUT-4 and -8 translocation to the atrial cell surface will be regulated by insulin and impaired during insulin-dependent diabetes. GLUT protein content was measured by Western blotting in healthy cardiac myocytes and type 1 (streptozotocin-induced, T1Dx) diabetic rodents. Active cell surface GLUT content was measured using a biotinylated photolabeled assay in the perfused heart. In the healthy atria, insulin stimulation increased both GLUT-4 and -8 translocation to the cell surface (by 100% and 240%, respectively, P<0.05). Upon insulin stimulation, we reported an increase in Akt (Th308 and s473 sites) and AS160 phosphorylation, which was positively (P<0.05) correlated with GLUT4 protein content in the healthy atria. During diabetes, active cell surface GLUT-4 and -8 content was downregulated in the atria (by 70% and 90%, respectively, P<0.05). Akt and AS160 phosphorylation was not impaired in the diabetic atria, suggesting the presence of an intact insulin signaling pathway. This was confirmed by the rescued translocation of GLUT-4 and -8 to the atrial cell surface upon insulin stimulation in the atria of type 1 diabetic subjects. In conclusion, our data suggest that: 1) both GLUT-4 and -8 are insulin-sensitive in the healthy atria through an Akt/AS160 dependent pathway; 2) GLUT-4 and -8 trafficking is impaired in the diabetic atria and rescued by insulin treatment. Alterations in atrial glucose transport may induce perturbations in energy production, which may provide a metabolic substrate for atrial fibrillation during diabetes.

## Introduction

Diabetes mellitus is a serious metabolic disorder affecting 387 million people worldwide [1; 2]. Diabetes has now reached epidemic levels and has been identified as the 7^th^ leading cause of death in the USA [2; 3]. Hyperglycemia, the hallmark of diabetes, results from an impaired glucose uptake due to a lack of insulin production by pancreatic beta cell (type 1) or lack of insulin action (type 2). Diabetes results in multiple organ dysfunction including cardiomyopathy, coronary artery disease and atrial fibrillation [4–7].

Glucose is a major energy substrate for the heart, which generates ~30% of its total energy from glucose oxidation during physiological condition [8]. Therefore, cardiac glucose uptake and utilization is crucial for proper cardiac function. This is germane to the fact that the atria, which is the pacemaker of the heart, significantly contributes to the overall cardiac function. Although the rate of glucose utilization in the heart is greater than in other tissue, little is known about glucose metabolism in the atria during both healthy and disease states [9]. Glucose transport into the cell is the rate limiting step of glucose utilization and is regulated by a family of membrane proteins known as Glucose Transporters (GLUTs) [10]. Although GLUT4 (from the class I of GLUTs) is the main cardiac isoform (approximately 70% of the total cardiac GLUTs), recent evidence suggests that GLUT8, one of the most recently discovered isoforms in the class III, is also expressed in the heart [11–14]. The GLUT8 mRNA expression is reported to be the highest (i.e., ~7% of total GLUTs) in the murine left ventricle, following GLUT4 and GLUT1 [15]. In addition, it has been reported that there was a significant upregulation of GLUT8 protein expression in the left ventricle of GLUT4 knock out mouse [15]. However, there is no study that relatively quantifies the abundance of GLUT8 protein expression in the heart. In addition, although GLUT8 has been reported to be an insulin-dependent isoform in blastocysts [14], its functional role in the myocardium is yet to be determined. Whereas some other isoforms have been referred to as basal GLUTs located primarily at the cell surface (i.e., GLUT1, GLUT12), the translocation of the main GLUT protein, GLUT4, from an intracellular sequestration inactive site to the plasma membrane (active site) is largely regulated by insulin-dependent processes, although other factors can also alter myocardial glucose transport [16; 17]. Importantly, GLUT4 trafficking has been shown to precede glucose transport in insulin-sensitive tissues [18–20]. In skeletal muscle, following insulin stimulation, activation of IRS-1 protein induces the activation of several kinases, which in turn recruit the pivotal serine/threonine protein kinase, namely, Akt. The activated Akt phosphorylates a downstream protein AS160 (an Akt substrate protein of 160 kDa) which is essential for the exocytosis of GLUT4 to the plasma membrane [21]. Although the downstream insulin signaling pathway has been studied in the skeletal muscle, it is yet to be characterized in the heart, especially in the atria. Better understanding of the role and regulation of glucose transport in the healthy and diabetic atria will give novel insights in understanding the pathophysiology of diabetes and its associated cardiovascular complications.

It has been well documented that there is a regional heterogeneity between the atria and ventricles regarding their structure and function. Therefore, one could hypothesize that the differences in contraction and flow distribution pattern between atria vs. ventricle may also contribute to the regional metabolic heterogeneity [22; 23]. However, the majority of the studies that have investigated cardiac energetics have studied global or left ventricular changes [24; 25]. A recent study from our group suggested an impairment of glucose transport in the atria of a canine model of heart failure [22]. However, in this model, heart failure was induced by rapid ventricular pacing, which may have had a profound effect on metabolism. Therefore, the role of glucose uptake and utilization in the healthy and diseased atria has received little attention. In the current study, we hypothesized that 1) the major cardiac GLUT isoform, GLUT4, and the novel GLUT isoform, GLUT8, will be regulated by insulin in the atria and 2) GLUT translocation will be impaired during diabetes. The enclosed study provides novel insights into the expression and regulation of insulin-sensitive GLUTs in the atria, which could lead to a better understanding of the uptake of glucose, a major metabolic substrate for the heart.

## Materials and Methods

### Animals

Healthy and insulin-deficient type 1 diabetic (T1Dx) FVB/N mice were used, as previously described [24–27]. Briefly, type 1 diabetes was induced at 10–12 weeks of age by 3 consecutive doses of freshly prepared steptozotocin (STZ, 65–95 mg/kg IP every 48 hours, diluted in citrate buffer), while the control group received placebo injection (citrate buffer). All mice were fed a standard diet to maintain body weight (10% kcal from fat) for the duration of the study. Diabetes was confirmed by measuring venous blood glucose concentration (facial vein) at baseline and every week for both groups, using a glucometer (Bayer Contour, Tarrytown, NY) on mice fasted overnight for 8 hours. Fasted plasma serum insulin was measured in duplicate using an ELISA kit (Millipore). Eight weeks after the induction of diabetes, animals were deeply anesthetized and the heart was rapidly removed and underwent a retrograde perfusion using a Langendorff apparatus. All the procedures of this study were approved by the Oklahoma State University Institutional Animal Care and Use Committee (#VM-12-3).

### Myocyte isolation

Expression of GLUTs and the major proteins involved in the insulin signaling pathway was measured in isolated atrial myocytes. Atrial and ventricular myocytes were obtained by retrograde perfusion of the heart from healthy adult rats using a Langendorff apparatus and enzymatic digestion with collagenase (Worthington labs, NJ), as previously described [24–28]. A minimum yield of 70% live cells were considered acceptable. Myocytes were incubated with or without (basal) insulin (0.01 μM, for 1 hour at room temperature).

### Protein extraction

Total and crude extracts of membrane-enriched protein lysates of atrial and ventricular myocardium were prepared as previously described [24–27]. Briefly, total protein lysates from fresh atrial and ventricular myocytes/tissue were collected by incubating the myocytes/tissue with lysis buffer (RIPA, Thermo fisher Scientific). Samples were centrifuged at 3000 g for 25 min, and the supernatant was stored at − 80°C until further analysis. Crude membrane protein extracts were collected from frozen tissue samples that were homogenized in buffer containing (mM): sucrose 210, NaCl 40, EDTA 2, HEPES 30, and protease inhibitor (Sigma, St. Louis, MO). The homogenate was incubated with sodium pyrophosphate 58 mM and KCl 1.17 mM. Crude membranes were then recovered by centrifugation at 100,000 *g* for 90 min at 4°C. Pellets were re-suspended with a cell lysis buffer (RIPA, Thermofisher Scientific). Samples were centrifuged at 3000 g for 25 min, and the supernatant was stored at − 80°C until further analysis.

### Western immunoblotting

Equal amounts of protein (5–20 μg) were resolved in an 8–12% SDS-polyacrylamide gel and electrophoretically transferred (BioRad) to a polyvinyl-idine fluoride membrane (Biorad), as previously described [22; 24–27; 29; 30]. After blocking (1–5% non-fat dry milk or 2% goat serum albumin), membranes were incubated with optimally diluted primary antibodies overnight (polyclonal rabbit anti-human GLUT4, 1:750, AbD Serotec 4670–1704; polyclonal rabbit anti-human GLUT8, 1:500, Bioss bs-4241R; monoclonal rabbit anti-mouse total Akt, 1:1000, Cell Signaling 4061; monoclonal rabbit anti-human phosphorylated Akt s473, 1:1000, Cell Signaling 4060; monoclonal rabbit anti-mouse phosphorylated Akt Th308, 1:1000, Cell Signaling 2965; monoclonal rabbit anti-human total AS160, 1:1000, Cell Signaling 2670 and polyclonal rabbit anti-human phosphorylated AS160, 1:1000, Cell Signaling 9611) followed by a 1 hour incubation of appropriate secondary antibodies conjugated to horseradish peroxidase (for total and phosphorylated Akt and AS160, Cell Signaling 7074, 1:2000, polyclonal goat anti-rabbit; for others, GE Healthcare NA934V, polyclonal donkey anti-rabbit). Primary antibodies were chosen based on their 100% sequence homology with the protein of interest in rodents, and validated against a positive control (i.e., tissue, peptide). Antibody-bound transporter proteins were quantified by enhanced chemiluminescence reaction (KPL) and autoradiography. Band density and molecular weight were quantified using GelPro Analyzer (Media Cybernetics). The data was expressed relative to appropriate controls. Equal protein loading was confirmed by reprobing each membrane with Calsequestrin monoclonal IgG (Thermo-Scientific PA1-903, 1:2500, polyclonal rabbit anti-dog).

### Quantification of GLUT translocation to the cell surface

Following a 1 hour Langendorff perfusion (with and without 0.7 nm insulin), both healthy and T1Dx atria and ventricles were photolabeled with the cell surface impermeant biotinylated bis-glucose photolabeling reagent (bio-LC-ATB-BGPA, 300 μM, Toronto Research Chemicals, ON, Canada), of which the hexose group interacts specifically with the extracellular binding site of GLUT. The photolabeled reagent was infused into the intact heart through the aorta before cross-linkage to cell surface GLUTs using a Rayonet photochemical reactor (340 nm, Southern New England UV), as previously described [24; 25; 31]. Protein extraction was immediately followed with homogenization and ultracentrifugation (227,000 g, 50 min at 4°C). Recovery of photolabeled (cell surface) GLUTs from total cardiac membranes (200 μg) was achieved using streptavidin isolation (bound to 6% agarose beads) to facilitate separation of non-cell surface GLUTs ("unlabeled" or intracellular fraction that remains in the supernatant) from cell surface GLUTs ("labeled" or sarcolemmal fraction). The labeled GLUTs were then dissociated from the streptavidin by boiling in Laemmli buffer for 30 min prior to SDS-PAGE and subsequent immunoblotting with GLUT antibody. Proteins from the labeled fraction were quantified by densitometry relative to the positive control, as previously described [24; 25; 29; 30].

### Statistical analysis

Normality and homogeneity of data were tested using Shapiro-wilk and Levene test, respectively. Differences between means were assessed using Student’s t-tests, one or two-way analysis of variance (treatment and/or tissue) for *in vitro* measurements, as appropriate. Repeated measured 2 way ANOVA was performed with Student Newman Keuls post-hoc test for the *in vivo* measurements. If not normally distributed, the data was analyzed with a Mann-Whitney or Friedman test. Correlations were analyzed by linear regression. Statistical significance was defined as P<0.05. Data are presented as mean ± SE.

## Results

### Regional heterogeneity of GLUT in the healthy myocardium

**Fig 1:**
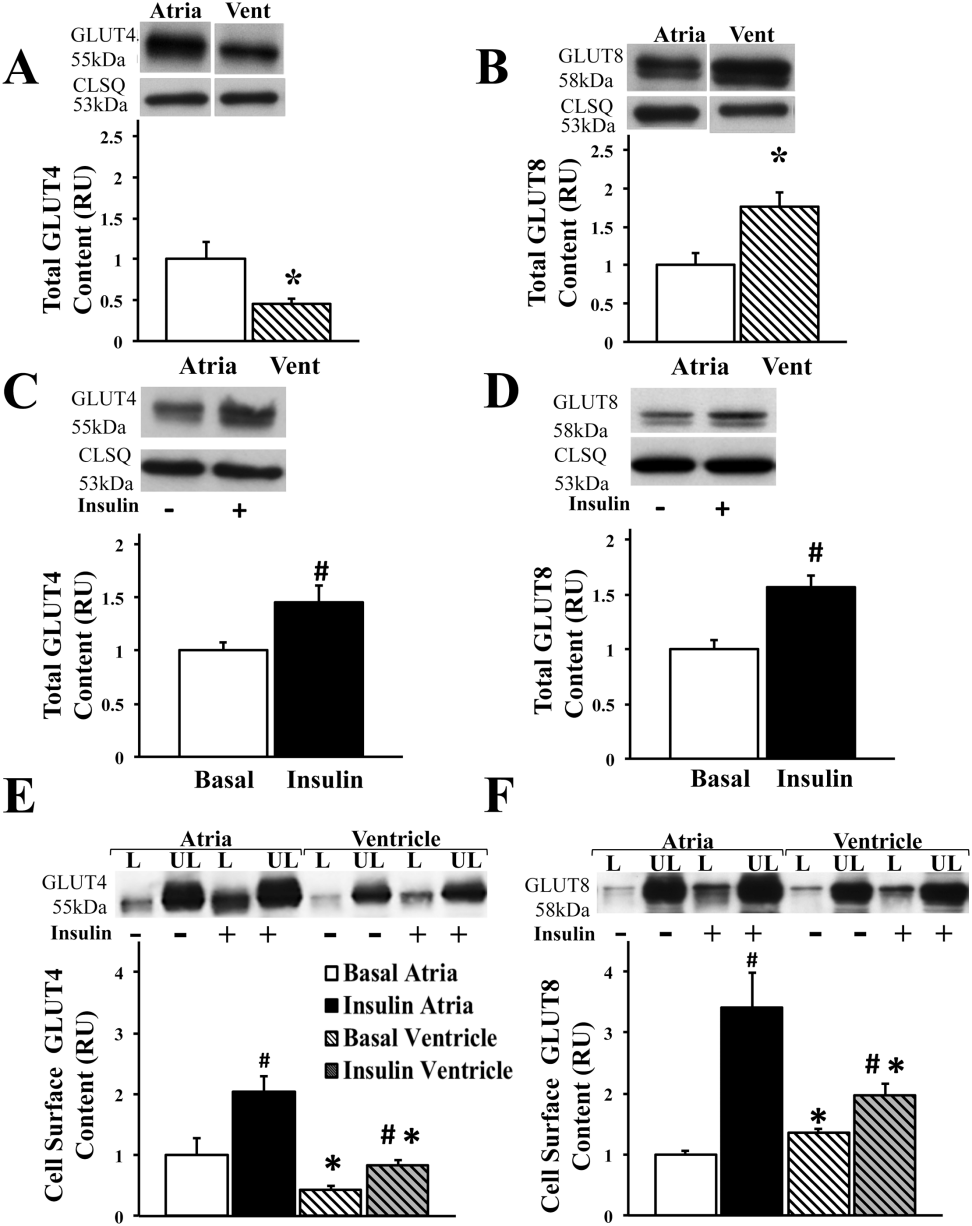
Regional heterogeneity of the insulin-sensitive GLUT4 and GLUT8 in the healthy myocardium. **Higher total protein expression of A) GLUT4 and lower B) GLUT8 content in the healthy atrial compared to ventricular myocytes.** Top panels: representative Western blot from total lysate of isolated rat myocytes; calsequestrin (CLSQ) was used as a loading control; representative bands were obtained from the same membrane. Bottom panels: Mean ± SE of total GLUT protein content (values expressed relative to atria; n = 6/group); * P<0.05 vs. atria. GLUT: glucose transporters. **Insulin stimulation increases C) GLUT4 and D) GLUT8 protein content** in the healthy atrial myocytes. Top panels: representative Western blot from total lysate of isolated rat myocytes, calsequestrin (CLSQ) was used as a loading control. Bottom panels: Mean ± SE of total GLUT protein content (values expressed relative to basal atria; n = 8-11/group); # P<0.05 vs. basal. **Insulin stimulates E) GLUT4 and F) GLUT8 trafficking to the atrial and ventricular cell surface.** Top panels: representative Western blot. Bottom panels: Mean ± SE of cell surface GLUT protein content (values expressed relative to labeled basal atria; n = 3-4/group); # P<0.05 vs. basal; *P<0.05 vs. atria. Methods: Cell surface GLUT measured using biotinylated photolabeling technique in the intact perfused mouse heart. L: Labeled (cell surface fraction); UL: Unlabeled (intracellular fraction).

Since glucose transport has not been well characterized in the atria, we first quantified total GLUT-4 and -8 protein content in the healthy myocardium. Our results indicated a significant regional heterogeneity of both the GLUTs in the atria vs. the ventricle. Total GLUT4 protein content was significantly greater in the isolated rat atrial myocytes compared to the ventricular myocytes (Fig 1A). In contrast, there was significantly greater GLUT8 content in the healthy ventricle compared to the atria (Fig 1B). In order to investigate the insulin sensitivity of the GLUTs in the healthy atria, we then measured protein content of GLUT-4 and -8 upon insulin stimulation in isolated rat atrial myocytes. Our results indicated an increase in total GLUT-4 and -8 total protein content (by 44% and 60% respectively, P<0.05) upon insulin stimulation (Fig 1C and 1D). In order to measure GLUT translocation to the cell surface, the rate limiting step in glucose uptake, we used the biotinylation photolabeled assay in the intact perfused mouse heart to reliably quantify the proportion of active cell surface GLUTs at the atrial and ventricular cell surface (Fig 1E and 1F). Our results confirmed the regional heterogeneity of GLUT4 and GLUT8 in the atrial and ventricular cell surface, showing significantly greater cell surface GLUT4 content in the atria and greater cell surface GLUT8 content in the ventricle respectively (Fig 1E and 1F). To further determine whether GLUT-4 and -8 are insulin-sensitive, the biotinylation photolabeled assay was performed in the heart perfused with or without (basal) physiological concentration of insulin. Upon insulin stimulation, there was significant increase in cell surface GLUT-4 and -8 content in both the atrial (by 100% and 240% vs. basal condition, respectively, P<0.05) and ventricular cell surface (by 97% and 40% vs. basal condition, respectively, P<0.05), indicating that both GLUT-4 and -8 are insulin-sensitive in the atria and ventricle. Interestingly, the response to insulin was greater in the atria compared to the ventricle, with an increase in GLUT8 trafficking by 240% vs. 40% (compared to basal condition), respectively (P<0.05).

### Analysis of downstream insulin signaling pathway in the healthy atria

**Fig 2:**
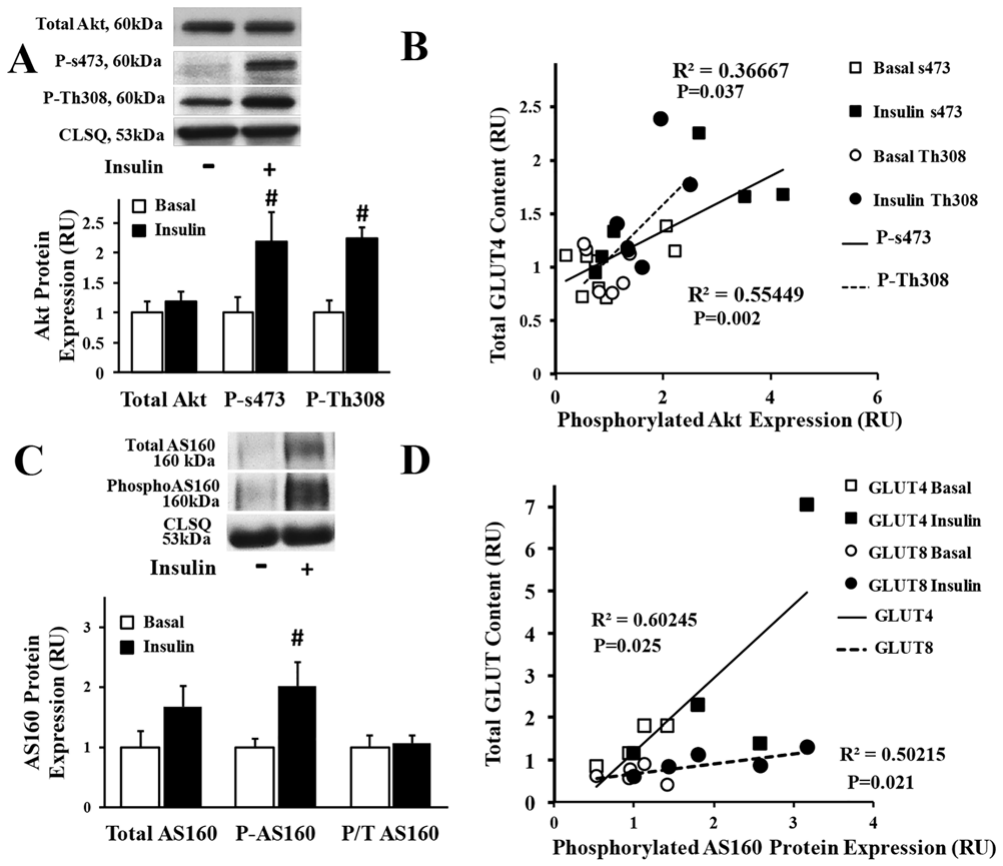
Analysis of the downstream insulin signaling pathways in the healthy atria. **A) Insulin stimulates phosphorylation of Akt at s473 and Th308 site** in atrial myocytes. Top panel: representative Western blot from total lysate of isolated rat atrial myocytes incubated with (0.01μM) and without (basal) insulin; calsequestrin (CLSQ) was used as a loading control. Bottom panel: Mean ± SE of protein expression (values expressed relative to basal; n = 5/group); # P<0.05 vs. basal. **B) Significant linear positive linear correlation between Akt phosphorylation (at s473 and Th308 site**) **and GLUT4 expression** in the healthy atria. **C) Insulin stimulates phosphorylation of AS160** in atrial myocytes. Top panel: representative Western blot from total lysate of isolated rat atrial myocytes; calsequestrin (CLSQ) was used as a loading control. Bottom panel: Mean ± SE of protein expression (values expressed relative to basal; n = 6-8/group); # P<0.05 vs. basal. **D) Significant linear correlation between AS160 phosphorylation and GLUT-4 and -8 expression** in the healthy atria.

In order to investigate the mechanisms regulating the translocation of GLUT-4 and -8 to the atrial cell surface, we then explored the downstream insulin signaling pathway, by incubating rat atrial myocytes with and without (basal) insulin. We reported a significant increase in the phosphorylation of Akt (at s473 and Th308 sites) and AS160 upon insulin stimulation (Fig 2A and 2C). No significant change was observed when phospho AS160 (Fig 2C) was compared to total AS160. In addition, we reported a significant positive linear correlation between total GLUT4 protein content and phosphorylation of Akt (Fig 2B), as well as between total GLUT protein content and AS160 activation (Fig 2D).

### Alteration in GLUT expression and trafficking during type 1 diabetes

**Fig 3:**
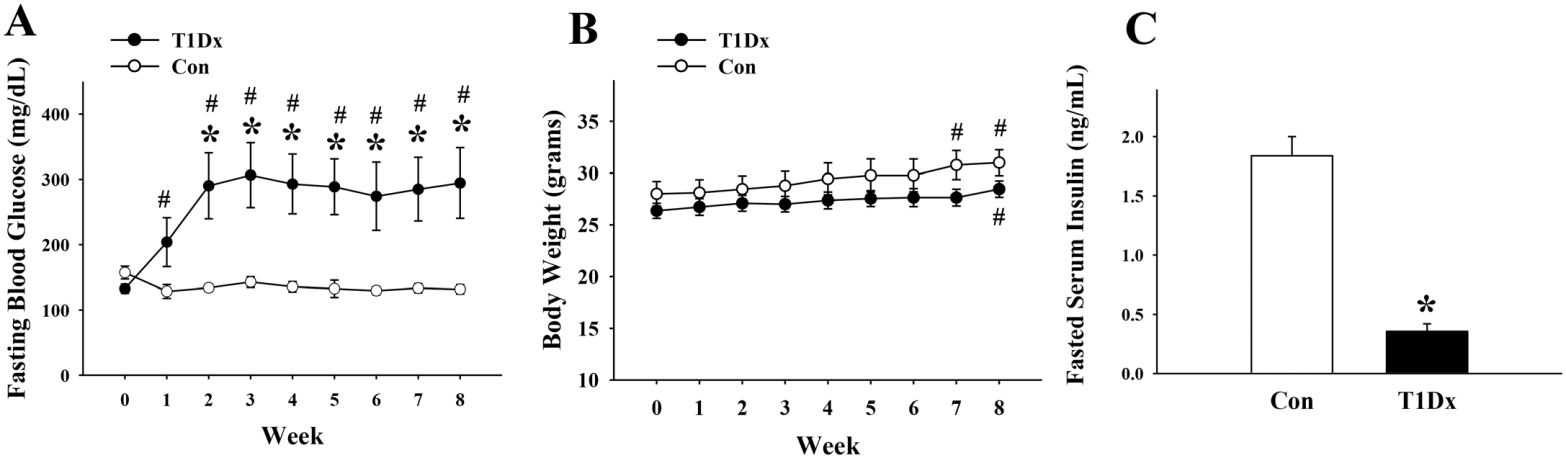
Validation of the insulin-deficient (type 1) diabetic animal model. **A) Mean ± SE venous blood glucose concentration** obtained at baseline and up to 8 weeks in type 1 diabetic (T1Dx) and age-matched control (Con) mice (n = 9-11/group). **B) Mean ± SE body weight** obtained at baseline and up to 8 weeks after induction of type 1 diabetes (n = 9-11/group). **C) Mean ± SE serum insulin concentration** obtained at 8 weeks after induction of type 1 diabetes (n = 6-8/group). T1Dx: type 1 diabetic; Con: control; *P<0.05 vs. control; # P<0.05 vs. baseline.

In order to investigate alterations in glucose transport during diabetic conditions, we used an insulin-deficient diabetic animal model (T1Dx). As expected, within 1 week after injection, STZ-treated animals developed hyperglycemia (i.e., [glucose]>200 mg/dl), which persisted throughout the experimental period; while the control group remained euglycemic (Fig 3A). There was no difference in body weight between groups, although both control and diabetic mice weighted more (P<0.05) at the end of the experimental period (Fig 3B). In addition, serum insulin level was significantly lower in diabetic compared to control mice, confirming that STZ administration destroyed the beta cells of the pancreas (Fig 3C).

**Fig 4:**
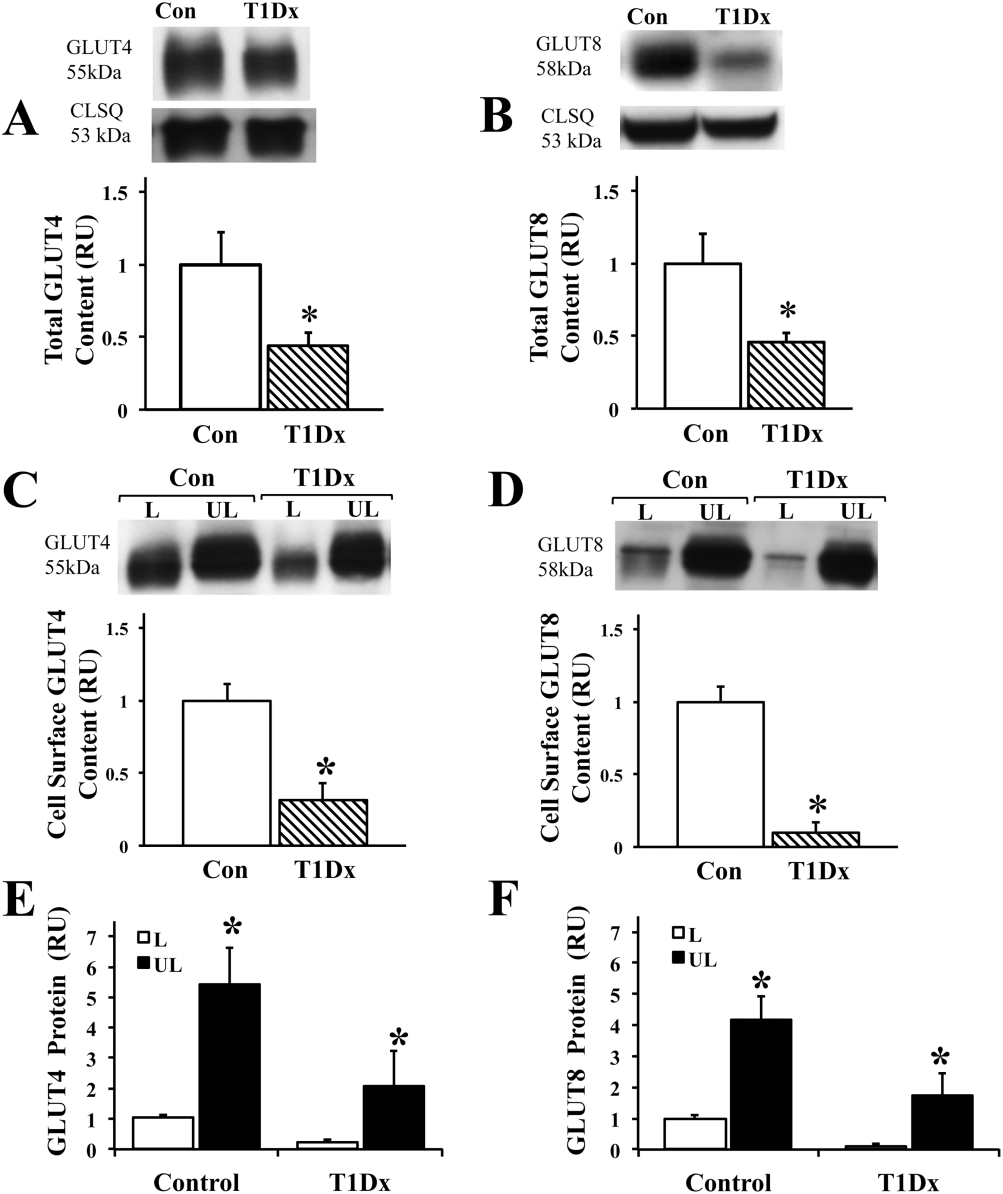
Alteration of the trafficking of the insulin-sensitive GLUTs in the diabetic atria. **Decreased atrial A) GLUT4 and B) GLUT8 content during type 1 diabetes** (T1Dx). Top panels: representative Western blot from crude membrane extract of perfused mouse atria, calsequestrin (CLSQ) was used as a loading control. Bottom panels: Mean ± SE of GLUT protein content (values expressed relative to control; n = 7-8/group). **Type 1 diabetes decreased C) GLUT4 and D) GLUT8 trafficking to the atrial cell surface.** Top panels: representative Western blot. Bottom panels: Mean ± SE of cell surface GLUT protein content (values expressed relative to control; n = 4-5/group). **Majority of E) GLUT4 and F) GLUT8 is intracellular under basal conditions** (mean ± SE, values expressed relative to control labeled, n = 5/group). Methods: Intact perfused mouse hearts were photolabeled with bio-LC-ATB-BGPA to determine the amount of cell surface (L: labeled fraction) and intracellular (UL: unlabeled fraction) content. T1Dx: type 1 diabetic; Con: control; * P<0.05 vs. control.

We then quantified GLUT-4 and -8 total protein expressions in a crude membrane enriched extract of the mouse atria (Fig 4A and 4B). Our results indicated a significant decrease in GLUT-4 and -8 total protein content in the type 1 diabetic group compared to healthy controls. To accurately differentiate plasma membrane-associated GLUT4 from intracellular membrane-associated GLUT4, we used the biotinylated assay in the intact perfused atria. We observed a down-regulation of atrial cell surface GLUT-4 and -8 by 70% and 88%, respectively, in diabetic animals compared to healthy controls (P<0.05, Fig 4C and 4D). Furthermore, we quantified the proportion of GLUT located at the cell surface compared to the intracellular pool for both GLUT-4 and -8 (Fig 4E and 4F). As expected, under basal conditions, GLUT4 cell surface fraction was 26% and 17% of the intracellular fraction during healthy and diabetic condition, respectively (P<0.05). Similarly, under basal conditions, GLUT8 cell surface fraction was 25% and 5% of the intracellular fraction during healthy and diabetic condition, respectively (P<0.05, Fig 4E and 4F). Therefore, our results demonstrated a significant down-regulation of total protein expression and trafficking to the atrial cell surface of GLUT-4 and -8 during type 1 diabetic condition.

### Role of insulin in regulating GLUT trafficking to the atrial cell surface during type 1 diabetes

**Fig 5:**
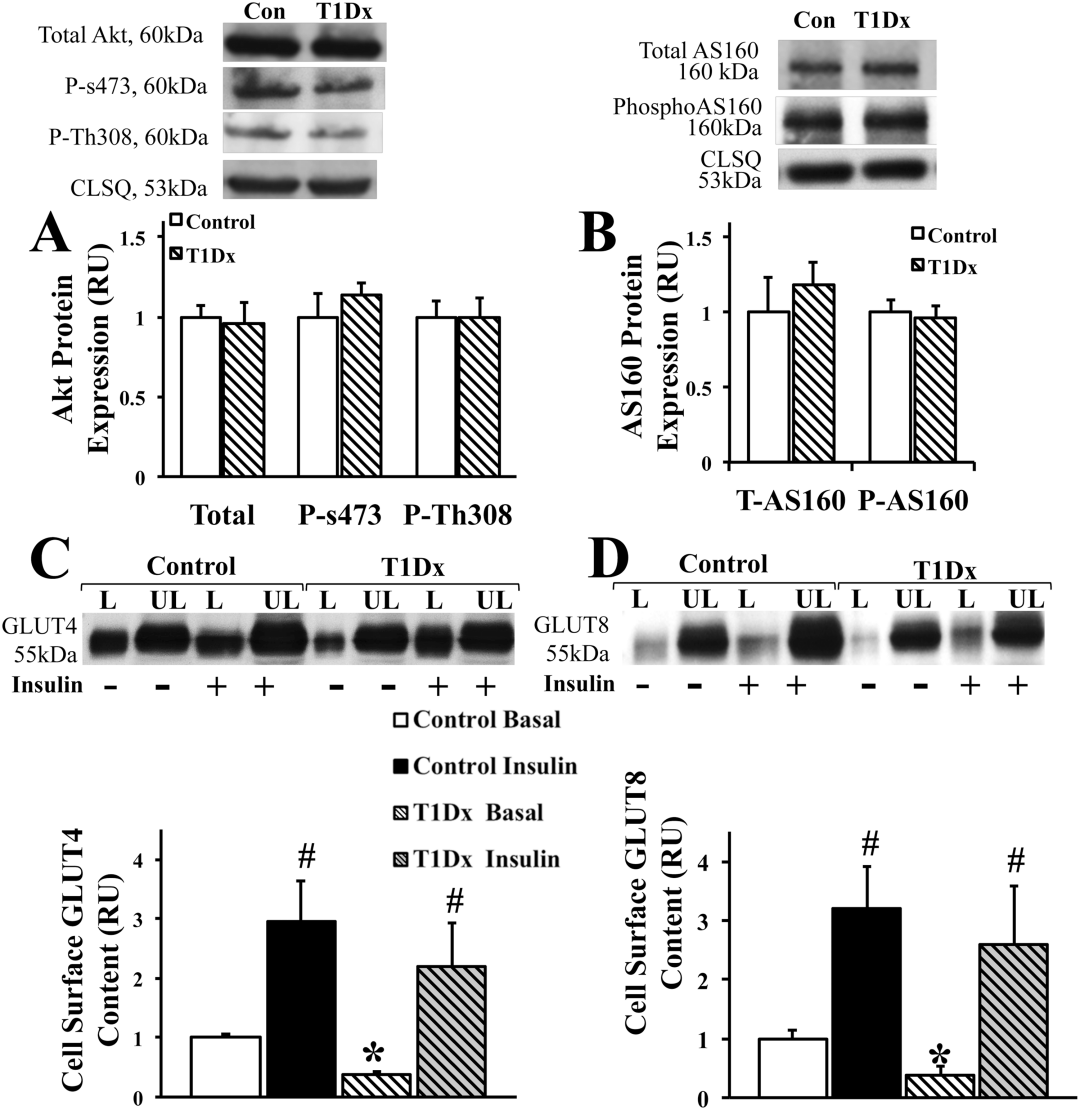
Intact insulin signaling pathway in the atria of insulin-deficient diabetic animals. **Type 1 diabetes (T1 Dx) did not alter A) Akt or B) AS160 phosphorylation in the atria**. Top panels: representative Western blot from total lysate of mouse atria; calsequestrin (CLSQ) was used as a loading control. Bottom panels: Mean ± SE of protein expression (values expressed relative to control; n = 4-5/group). **Insulin stimulates C) GLUT4 and D) GLUT8 trafficking to the atrial cell surface in type 1 diabetic (T1 Dx) subjects**. Top panels: representative Western blot. Bottom panels: Mean ± SE of cell surface GLUT protein content (values expressed relative to control basal labeled; n = 4-6/group). Methods: Intact mouse hearts were perfused with and without insulin, and photolabeled with bio-LC-ATB-BGPA to determine the amount of cell surface (L: labeled fraction) and intracellular (UL: unlabeled fraction) content. T1Dx: type 1 diabetic; *P<0.05 vs. control; # P<0.05 vs. basal.

Despite intensive research during the last few decades (primarily in skeletal muscle and adipose tissue), the pathogenic cause of altered glucose transport during diabetes remains elusive. Therefore, we further analyzed the possible alteration in the downstream insulin signaling pathway under diabetic conditions, to understand whether or not the impairment in GLUT trafficking can be attributed to the defective insulin signaling pathway. Interestingly, our results indicated no alteration in the phosphorylation of either Akt or AS160 in diabetic subjects (Fig 5A and 5B) compared to the controls. Using the biotinylation assay in the intact perfused mouse atria, we further observed a rescued trafficking of both GLUT-4 and -8 to the atrial cell surface upon *in vitro* insulin stimulation in the diabetic atria (Fig 5C and 5D). Collectively, the data suggested an intact insulin signaling pathway in the T1Dx atria.

## Discussion

Although the heart is one of the main organs to utilize glucose as a metabolic substrate, very little is known about atrial glucose metabolism in both healthy and diseased conditions. Our data demonstrates that 1) a regional heterogeneity exists between GLUT4 and 8 expression in atria vs. ventricle; 2) both GLUT-4 and -8 are insulin-sensitive in the healthy atria; 3) diabetes impaired GLUT-4 and -8 trafficking to the atrial cell surface; and 4) insulin stimulation rescued GLUT translocation to the atrial cell surface during type 1 diabetes.

In order to sustain its high energy demand, the rate of glucose utilization in the heart is greater than in skeletal muscle, adipose tissue and lung, despite the ability of the myocardium to use other substrates (i.e., fatty acids, lactate, ketone bodies and amino acids) [9]. Glucose entry from the blood stream into the cell is the rate-limiting step in glucose utilization and occurs primarily via a group of facilitative glucose transporters (GLUT) [10]. Despite the crucial role of the atria as a pacemaker of the heart, studies on the role of glucose transport and utilization in the atria have been scarce. The majority of the studies that have investigated cardiac energetics have studied the global or left ventricular changes [23; 24; 32–35]. Due to the relatively small size of atria, specifically in rodent models (i.e., <30 mg), it is difficult to study membrane trafficking of GLUTs and, to our knowledge, we are the first to apply the biotinylation assay technique to the study of GLUTs in the atria. Please note that we previously reported that GLUT trafficking upon insulin stimulation is comparable between isolated rat ventricular myocytes and intact mouse ventricle [25]. Using the photolabeled technique in the intact perfused mouse heart, we demonstrated that GLUT4 translocated to the atrial cell surface upon insulin stimulation, similar to findings reported in the ventricle [24; 25]. We further observed in the current study a greater active cell surface GLUT4 content in the healthy atria compared to the ventricle under basal and insulin stimulated conditions. The finding of this study is consistent with results from Ware et al. [22], who demonstrated regional pattern of total GLUT4 expression in the chambers of the healthy myocardium. The mechanisms underlying this regional heterogeneity are not well elucidated. In order to sustain the faster contraction rate, atrial myocytes possess unique electrophysiological properties, including faster calcium release from the sarcoplasmic reticulum (SR), greater inositol 1,4,5-trisphosphate (IP3) content and greater calcium buffering abilities [36]. Since SR calcium handling regulates GLUT4 trafficking to cell surface in the myocardium [25], one could speculate that these well-known differences in calcium homeostasis could explain the greater cell surface GLUT4 expression in the atria compared to the ventricle. This regional heterogeneity further underscores the importance to specifically study glucose metabolism in the tissue of interest and the fact that one cannot extrapolate findings from the ventricle to the atria.

Class I transporter GLUT4, the most extensively studied insulin-sensitive GLUT, is predominantly expressed in the adult heart (~70% of total myocardium GLUTs) and is thought to be responsible for insulin-stimulated glucose uptake [16]. However, in a study conducted by Katz et al., it has been reported that GLUT4 null mice do not develop hyperglycemia, suggesting that other GLUT isoforms could be involved in the regulation of whole-body glucose homeostasis [37]. GLUT8, one of the most recently discovered GLUT isoforms and member of the lesser known Class III, has been shown to be present in substantial quantities in the cardiac tissue and thus, may play a significant role in glucose uptake [15]. However, whether or not GLUT8 is primarily located at the cell surface or in intracellular compartment is unclear. Although GLUT8 has been reported to be an insulin-dependent isoform in blastocysts and hepatocytes [13; 14], its role in the heart has not been determined. Similar to GLUT4, our data demonstrated that GLUT8 translocates from an intracellular pool to the cell surface of the healthy myocardium upon insulin stimulation using the biotinylation assay. In addition, our findings also demonstrate that similar to GLUT4, GLUT8 is mostly intracellular under basal condition. This is in agreement with previous studies that reported that GLUT8 carries a NH_2_-terminal di-leucine motif that directs the protein to an intracellular localization and has the potential to translocate to the plasma membrane [15]. Collectively, these data suggest that GLUT8 is a novel insulin-sensitive GLUT in the heart. Interestingly, in the present study, insulin stimulation increased GLUT8 translocation to the cell surface in the atria to a greater extent than in the ventricle, suggesting that GLUT8 primarily contributes to insulin-stimulated glucose uptake in the atria. Due to the diversity of the insulin-sensitive GLUTs, characterization of the relative abundance and role of these novel GLUT isoforms (primarily from the Class III) in the heart is an important step towards understanding how these GLUTs contribute towards maintaining myocardial glucose transport.

In all the insulin-sensitive tissues (i.e., striated muscle and adipose tissue), insulin signaling via PI3K/Akt pathways plays a key role in cardiac glucose uptake. As insulin binds to its receptor, the downstream insulin signaling pathway is activated, resulting in the phosphorylation of protein kinase Akt at threonine 308 (Th308) residues, which is located in the kinase domain of the Akt activation loop. The full activation of Akt requires a further phosphorylation of serine 473 (s473) residues [38; 39]. It is well documented that the phosphorylation of both sites (s473 and Th308) are crucial for GLUT trafficking. The activated Akt in turn phosphorylates AS160, which is the most distal signaling protein that has been implicated in insulin mediated GLUT translocation and has emerged as a key regulator of GLUT trafficking [21]. Under basal conditions, AS160 retains GLUT vesicles at the intracellular inactive pool [39]. The phosphorylation of AS160 allows the activation of RabGTP, which initiates the signal transduction pathway that is necessary for the docking and diffusion of GLUT vesicles on the plasma membrane [21]. It is important to note that, the initiation of the signaling cascade by the activation of the insulin receptor not only increases GLUT4 exocytosis at the membrane, it also attenuates GLUT4 endocytosis causing an enhanced re-distribution of GLUT4 protein at the plasma membrane, facilitating cellular glucose uptake [40]. Although this pathway has been well investigated in skeletal muscle and adipose tissue, the downstream signaling pathway that regulates glucose transport in the atria remains to be characterized. Our findings indicated that insulin stimulation increased the phosphorylation of Akt (at both s473 and Th308 sites) and AS160 in cardiac myocytes. We further demonstrated that the activation of these major proteins involved in the downstream insulin signaling pathway was significantly correlated to the increased GLUT protein expression in the atria. Due to the small size of the rodent atria, both membrane trafficking measurements and quantification of cytosolic proteins were not technically possible to perform in the same sample. Nonetheless, collectively, these data suggest that the insulin signaling pathway regulates GLUT trafficking in the atria. In addition to Akt activating GLUT trafficking, there is an increasing evidence that Rac1 activation is essential for GLUT4 trafficking to the cell surface and insulin-stimulated glucose uptake in skeletal muscle [41–45]. In addition, Akt-independent signaling pathway through TUG (tether containing a UBX domain for GLUT4) has been reported to be essential in maintaining protein stability and trapping the GLUT4 containing storage vesicles in the Golgi matrix in skeletal muscle and adipose tissue [46–49]. Although cardiac GLUT trafficking may be regulated by similar Akt-dependent and Akt-independent pathways, additional studies are required to fully characterize the regulation of GLUT trafficking in the myocardium, including in the atria.

STZ administration destroys the beta cell of the pancreas and induces a hyperglycemic state. As such, it is a well-established experimental model of insulin-deficient (type 1) diabetes [24–26]. Although alterations of metabolic efficiency have been described in the ventricle of this animal model [24–26; 50], whether diabetes induces alterations in glucose metabolism in the diabetic atria is unknown. Our data demonstrated a decrease in total GLUT-4 and -8 protein content in the atria of STZ-induced diabetic animals. Further, using the biotinylated photolabeled assay, our data demonstrated a 70% and 88% decrease in cell surface GLUT-4 and -8 protein content in the diabetic atria, respectively. This finding is consistent with the previous studies that reported impaired GLUT4 translocation in the ventricle of type 1 diabetic rats [50]. In addition, the role and regulation of GLUT8 during insulin-deficient diabetic conditions have received scarce attention. Gorovits et al. reported that short-term STZ-induced diabetes significantly decreased GLUT8 mRNA levels in the liver and suggested that GLUT8 contributes to glucose homeostasis [13]. Similar quantification of GLUT mRNA expression in the atria could be an avenue for future investigations. In another study, Pitoli et al. reported the alteration of protein expression and disruption of subcellular distribution of GLUT8 in the hippocampus of the type 1 diabetic rat [51; 52]. However, to our knowledge, there is no study that has investigated the regulation of GLUT8 in the cardiac tissue, more specifically in the atria. Our findings demonstrate that diabetes induces an impairment in GLUT8 trafficking to the cell surface of the atria.

We further investigated whether the down-regulation of cell surface GLUT-4 and -8 protein expressions was attributed to a defective insulin signaling pathway and/or due to a lack of insulin production in the atria of type 1 diabetic subjects. We reported no significant impairment in the phosphorylation of Akt (both s473 and Th308) and AS160, suggesting that the downstream insulin signaling pathway is not altered. This finding is consistent with a study by Laviola and colleagues, who reported that there was no significant decrease in the phosphorylation of Akt at s473 and Th308 sites in the ventricle of STZ induced diabetic rats compared to controls [53]. To further confirm these findings, we stimulated the diabetic atria with and without insulin (at physiological concentration), and our results demonstrated that insulin stimulation rescued cell surface GLUT4 and 8 protein expression in the diabetic atria, suggesting that the down-regulation of both insulin-sensitive GLUTs was mostly attributed to the lack of insulin production (following beta cell destruction) rather than an existing defect in the insulin signaling pathway in the atria of type 1 diabetic animals.

## Conclusions

Using a cell surface biotinylation assay, we demonstrated that the major GLUT isoform, GLUT4, and the novel GLUT8 isoform are both insulin-sensitive transporters. Our data further suggested that GLUT translocation to the cell surface is modulated by the downstream insulin signaling pathway via Akt and/or AS160 phosphorylation. We further demonstrated that diabetes impairs the trafficking of both GLUT-4 and -8, which was rescued by insulin stimulation in the diabetic atria. These alterations in atrial glucose transport may induce perturbations in energy production and could provide a metabolic substrate for atrial fibrillation. Therefore, better understanding of the regulation of glucose transport may lead to the discovery of novel therapeutic targets for the treatment of cardiovascular complications associated with diabetes, including atrial fibrillation.
